# Cerebellar Repetitive Transcranial Magnetic Stimulation and Noisy Galvanic Vestibular Stimulation Change Vestibulospinal Function

**DOI:** 10.3389/fnins.2020.00388

**Published:** 2020-04-28

**Authors:** Akiyoshi Matsugi, Shinya Douchi, Rikiya Hasada, Nobuhiko Mori, Yohei Okada, Naoki Yoshida, Satoru Nishishita, Koichi Hosomi, Youichi Saitoh

**Affiliations:** ^1^Faculty of Rehabilitation, Shijonawate Gakuen University, Daito, Japan; ^2^Department of Rehabilitation, National Hospital Organization Kyoto Medical Center, Kyoto, Japan; ^3^Department of Rehabilitation, Nagahara Hospital, Higasiosaka, Japan; ^4^Department of Neuromodulation and Neurosurgery, Graduate School of Medicine, Osaka University, Osaka, Japan; ^5^Department of Neurosurgery, Graduate School of Medicine, Osaka University, Osaka, Japan; ^6^Faculty of Health Sciences, Kio University, Koryo, Japan; ^7^Neurorehabilitation Research Center, Kio University, Koryo, Japan; ^8^Institute of Rehabilitation Science, Tokuyukai Medical Corporation, Toyonaka, Japan; ^9^Kansai Rehabilitation Hospital, Toyonaka, Japan

**Keywords:** cerebellum, transcranial magnetic stimulation, H-reflex, vestibular, galvanic vestibular stimulation

## Abstract

**Background:**

The cerebellum strongly contributes to vestibulospinal function, and the modulation of vestibulospinal function is important for rehabilitation. As transcranial magnetic stimulation (TMS) and electrical stimulation may induce functional changes in neural systems, we investigated whether cerebellar repetitive TMS (crTMS) and noisy galvanic vestibular stimulation (nGVS) could modulate vestibulospinal response excitability. We also sought to determine whether crTMS could influence the effect of nGVS.

**Methods:**

Fifty-nine healthy adults were recruited; 28 were randomly allocated to a real-crTMS group and 31 to a sham-crTMS group. The crTMS was conducted using 900 pulses at 1 Hz, while the participants were in a static position. After the crTMS, each participant was allocated to either a real-nGVS group or sham-nGVS group, and nGVS was delivered (15 min., 1 mA; 0.1–640 Hz) while patients were in a static position. The H-reflex ratio (with/without bilateral bipolar square wave pulse GVS), which reflects vestibulospinal excitability, was measured at pre-crTMS, post-crTMS, and post-nGVS.

**Results:**

We found that crTMS alone and nGVS alone have no effect on H-reflex ratio but that the effect of nGVS was obtained after crTMS.

**Conclusion:**

crTMS and nGVS appear to act as neuromodulators of vestibulospinal function.

## Introduction

The vestibular system and cerebellum allow for postural control and adaptation to several physical environments in human daily life. The investigation of the function of vestibular, cerebellum, and functional connectivity of both is important for the improvement of rehabilitation protocols. Studies using electrical stimulation and magnetic stimulation have revealed these functions. Electrical stimulation to deep cerebellar nuclei induces excitatory and inhibitory postsynaptic potentials in vestibular neurons through polysynaptic pathways (Ito et al., 1970), and lesions in the cerebellum disturb long-term adaptive changes in vestibular reflexes in animal models (Miles and Eighmy, 1980; Ito, 1998). These findings provide evidence for the functional connectivity between the cerebellum and vestibular complex (Jang et al., 2018). This connectivity is established through avenues such as the fastigial nucleus and interposed and dentate nuclei (Delfini et al., 2000). Such connectivity also refers to cerebellar involvement in the modulation of the excitability of vestibular reflexes (Straka et al., 2016).

Transcranial magnetic stimulation (TMS) non-invasively induces action potentials in cortical neurons and inhibits contralateral corticospinal excitability when applied over the cerebellar hemisphere (Ugawa et al., 1995; Daskalakis et al., 2004; Grimaldi et al., 2014; Matsugi and Okada, 2017, 2020). Repetitive TMS (rTMS) applied over a cerebellar hemisphere changes cerebellar brain inhibition (CBI) (Popa et al., 2010), indicating that cerebellar TMS (crTMS) can stimulate certain cerebellar tissues and that crTMS can modulate the excitability of cerebellar outputs. TMS applied over the inion improves eye-head coordination (Nagel and Zangemeister, 2003); as this effect requires vestibule-ocular function, the aforementioned finding indicates that TMS applied over the medial cerebellum affects vestibule-ocular function. Furthermore, as the vestibular nuclei comprise the common center for both vestibulo-ocular and vestibulospinal function (Straka et al., 2016), cerebellar TMS may affect vestibulospinal function. Therefore, in this study, we used the rTMS over inion to stimulate the central cerebellum, investigating whether cerebellar stimulation affect the vestibulospinal function (first aim of this study).

To test vestibulospinal function, galvanic vestibular stimulation (GVS) can be used (Fitzpatrick and Day, 2004). The firing rate of primary vestibular afferents can be decreased by the anodal square-wave pulse GVS (sqGVS) and increased by the cathodal sqGVS (Kim and Curthoys, 2004). Direct recording demonstrates that this stimulation induces action potentials in the vestibulospinal tract of the spinal cord and motor responses in target muscles with short latency (Muto et al., 1995). Furthermore, sqGVS can induce body sway in standing individuals (Fitzpatrick et al., 1994; Day et al., 2002). Changes in the activities of muscles that maintain postural control can be measured by electromyography (EMG) and the Hoffman reflex (H-reflex) (Iles and Pisini, 1992; Britton et al., 1993; Ali et al., 2003; Matsugi et al., 2017; Matsugi, 2019), which reflect the excitability of the spinal motoneuron pool (Knikou, 2008). Applied to an individual in a static position unaffected by natural body sway, change in the range of joints, or background EMG activity, sqGVS modulates the excitability of the H-reflex in the soleus muscle (Kennedy and Inglis, 2001; Ghanim et al., 2009; Lowrey and Bent, 2009; Okada et al., 2018). These observations indicate that the H-reflex-modulation induced by sqGVS reflects changes in the excitability of the vestibulospinal response. Therefore, in this study, we used this method to test vestibulospinal function.

Galvanic vestibular stimulation is often used not only to test vestibulospinal function but also to improve it. To improve balance mediated via facilitation of vestibulospinal function, the square-wave pulse GVS has not been used and random noise GVS (nGVS) is often used recently. The nGVS reportedly improves body balance in adults irrespective of age as well as in patients with vestibular disorder (Wuehr et al., 2017; Fujimoto et al., 2018). The stochastic resonance of noise addition to non-linear systems inducing the change in plasticity of information processing in neural systems may change the threshold or excitability of the motor response by vestibular input (McDonnell and Ward, 2011). However, it is unclear whether nGVS induces changes in vestibulospinal response excitability. Therefore, we investigated whether nGVS modulates the vestibulospinal function, as estimated by the H-reflex-modulation induced by sqGVS (second aim of this study).

Furthermore, the cerebellum is involved in the plasticity of vestibular reflex excitability, because crTMS modulates the effect of intervention for increased vestibulo-ocular movement for dynamic gaze (Matsugi et al., 2019). Deep cerebellar nuclei and Purkinje fibers in the cerebellar gray matter project to the vestibular nucleus. Stimulation of the cerebellar surface induces postsynaptic inhibitory or excitatory postsynaptic potentials in vestibular nuclei (Ito et al., 1970). Low-frequency repetitive stimulation of cortical neurons induces the long-term depression of synaptic excitability (Huerta and Volpe, 2009). Therefore, we hypothesized that crTMS affects the vestibular modulation via interventions such as nGVS in addition to that of exercise. Therefore, as the third aim of this study, we investigated whether rTMS applied over the cerebellum influenced the effect of nGVS on vestibulospinal excitability.

In summary, in this study, we investigated whether crTMS alone and nGVS alone modulate vestibulospinal function estimated by the H-reflex modulation induced by sqGVS. Further, we investigated whether crTMS modulates the effect of nGVS on the H-reflex-modulation induced by sqGVS.

## Materials and Methods

### Participants

Fifty-nine healthy adults (mean age, 23.8 ± 4.5 years; 40 men) participated in this study. None of the participants had histories of epilepsy or other neurological diseases. The Ethics Committee of Shijonawate Gakuen University approved the experimental procedures (approval code: 29-4), and this study was conducted according to the principles and guidelines of the Declaration of Helsinki; written informed consent was obtained from all participants.

### General Procedure

**FIGURE 1:**
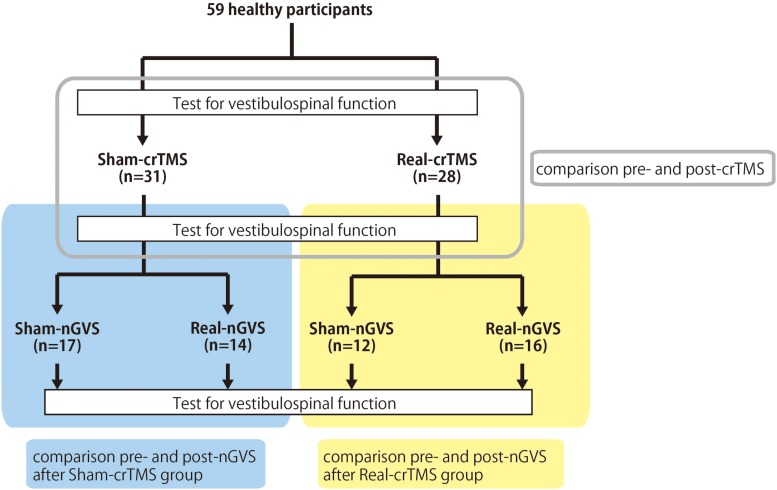
Experimental procedure and analysis. Fifty-nine healthy participants were allocated to either the sham-cerebellar repetitive TMS (crTMS) or real-crTMS groups. After crTMS, the participants were further allocated to either sham-noisy galvanic vestibular stimulation (nGVS) or real-nGVS groups. Tests for vestibulospinal function, including the H-reflex with/without conditioning square-wave pulse GVS, were conducted pre-crTMS, post-crTMS, and post-nGVS. In Analysis 1, the test parameters were compared between pre- and post-crTMS to elucidate the effect of crTMS on vestibulospinal function. In Analysis 2, the test parameters were compared between pre- and post-nGVS (post-sham-crTMS) to assess the effect of nGVS on vestibulospinal function. In Analysis 3, the test parameters were compared between pre- and post-nGVS (post-real-crTMS) to gauge the influence of crTMS on the effect of nGVS on vestibulospinal function. crTMS, repetitive transcranial magnetic stimulation; nGVS, noisy galvanic vestibular stimulation.

This study was conducted with a sham-controlled, double-blind design. The crTMS and nGVS conditions were blinded for participants and assessors when the assessments of vestibulospinal response were performed. Figure 1 presents the general procedures. All participants were allocated to either the sham-crTMS (*n* = 31) or real-crTMS groups (*n* = 28). After sham- or real-crTMS was completed, the participants in both groups were further subdivided into sham-nGVS and real-nGVS groups (after sham-crTMS: *n* = 17 and *n* = 14, respectively; after real-crTMS: *n* = 12 and *n* = 16, respectively), and then nGVS were conducted. Hence, all participants were randomly assigned to one of four groups, and three assessments of vestibulospinal function were conducted before (1st) and after crTMS (2nd) and after nGVS (3rd). If the participant experienced the sensation of the nGVS or could not endure the sqGVS in the test stimulation, the examination was ceased immediately.

Before the examination, we confirmed that the sqGVS did not produce sensations of pain or phosphine behind the eyes but did prompt body sway to the anodal side in participants standing with their eyes closed, feet together, and head facing forward (Fitzpatrick and Day, 2004) to test whether they were responders or non-responders to square-wave pulse GVS. In this timing, if the participant cannot endure square-wave pulses at 3 mA using all tests for vestibulospinal function, the participant did not participate in subsequent experiments. No participant responded to sqGVS at 3 mA, but four participants were excluded owing to the existence of unbearable pain (see “Results” section). We asked participants to report any sensation in response to nGVS; participants reporting sensation were excluded from analysis.

During the tests for vestibulospinal function (1st, 2nd, and 3rd), crTMS (sham or real) and nGVS (sham or real), participants lay down in the prone position while relaxing on the bed. The experiments were performed in the following order: (1) test for vestibulospinal function (1st test), (2) sham- or real-crTMS, (3) test for vestibulospinal function (2nd test), (4) sham- or real-nGVS, and (5) test for vestibulospinal function (3rd test).

### Conditioning Stimulation

#### Cerebellar Repetitive TMS (crTMS)

**FIGURE 2:**
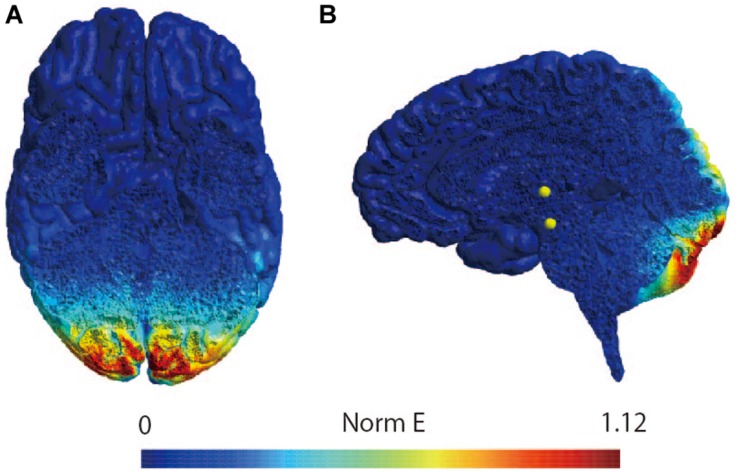
Simulation of the electrical field induced by cerebellar TMS. Simulation of the electrical field induced by TMS with butterfly coil using SimNIBS in horizontal slice **(A)** and sagittal slice **(B)**. The scale represents the magnitude of the electric field (Volts/meter) induced by TMS over the site at 1 cm under the inion.

The participants were instructed to lie down on a bed in the prone position. Because previous studies have shown that the figure-of-eight coil could stimulate the cerebellum (Haarmeier and Kammer, 2010; Popa et al., 2010; Tremblay et al., 2016), a magnetic stimulator (MagPro compact, MagVenture, Denmark) was used to deliver TMS to the cerebellum with a butterfly coil (MC-B70, MagVenture, Denmark) (Matsugi et al., 2019). The center of the coil’s junction was set at a distance 1 cm below the inion to stimulate the central region of the cerebellum (Zangemeister and Nagel, 2001; Nagel and Zangemeister, 2003; Jayasekeran et al., 2011; Hardwick et al., 2014; van Dun et al., 2017); prior research has demonstrated that stimulation from this position can modulate vestibular and ocular motor functions (Zangemeister and Nagel, 2001; Nagel and Zangemeister, 2003; Jenkinson and Miall, 2010). As previous studies have observed that an upward current applied to the cerebellum can effectively stimulate this region (Ugawa et al., 1995; Hiraoka et al., 2010; Matsugi et al., 2013), the coil was oriented such that the current therein was directed downward to deliver the upward current to the brain (van Dun et al., 2017). TMS intensity was set to 50% of the maximum stimulator output: the same setting as those employed by previous studies investigating cerebellar and vestibular functions (Zangemeister and Nagel, 2001; Nagel and Zangemeister, 2003; Haarmeier and Kammer, 2010; Jenkinson and Miall, 2010; van Dun et al., 2017; Matsugi et al., 2019). Because a 1-Hz crTMS reduces motor function and motor adaptation (Miall and Christensen, 2004; Jenkinson and Miall, 2010) the inter-stimulus interval was set at 1 s, and 900 pulses were applied (Fierro et al., 2007; Popa et al., 2010; Matsugi et al., 2019). In the stimulation condition, electrical field stimulation of the brain structures was performed with SimNIBS software (version 2.1.1) using default head models, biological tissue conductivity values included in the software, and the aforementioned parameters of the TMS using a butterfly coil (shown in Figure 2) (Thielscher et al., 2015). The coil was held at a 90° angle from the scalp over the inion when delivering sham-TMS (Hiraoka et al., 2010; Matsugi et al., 2013, 2014, 2019).

#### Noisy Galvanic Vestibular Stimulation (nGVS)

**FIGURE 3:**
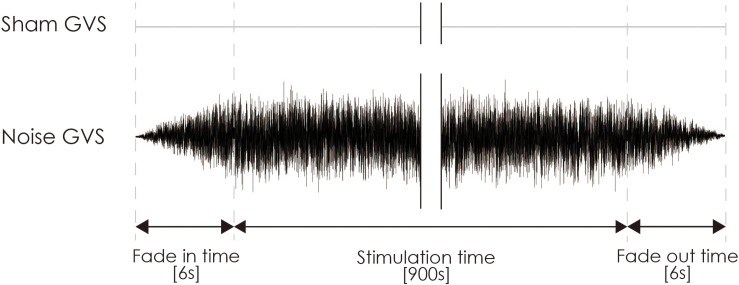
Typical waveform of nGVS and sham-nGVS. nGVS, noisy galvanic vestibular stimulation.

Noisy galvanic vestibular stimulation was performed as previously reported (Inukai et al., 2018). nGVS was delivered via Ag/AgCl surface electrodes (Blue Sensor EKG Snap Electrode, overall dimensions: 48 mm × 57 mm, Ambu, Baltorpbakken, Denmark) affixed to the right and left mastoid processes. A DC-STIMULATOR PLUS (Eldith, NeuroConn GmbH, Ilmenau, Germany) was used to deliver random noise galvanic stimulation to the primary vestibular nerve. For nGVS in the stimulation mode, “noise” was used, a random level of current was generated for every sample (sample rate, 1280 samples/s) (Moliadze et al., 2012; Inukai et al., 2018), and the intensity was set at 1 mA. Statistically, the random numbers were normally distributed over time, the probability density followed a Gaussian bell curve, and all coefficients featured a similar size in the frequency spectrum of this mode. A waveform was applied with 99% of the values between −0.5 and +0.5 mA, and only 1% of the current level was within ±0.51 mA. The stimulation time was set to 900 s, and the current was ramped up and down from 6 s before the stimulation to 6 s after its completion (Figure 3). Even if a slight sensation was felt, the participant was excluded from the experiment on account of the condition no longer being blind. For sham stimulation, direct current stimulation was applied at an intensity of 0 mA (sham-nGVS). If the participant sensed the stimulation of the real- or sham-nGVS, the participant was disqualified from further testing.

### Test for Vestibulospinal Function

To estimate the excitability of the vestibulospinal response, the H-reflex during short duration square-wave pulse GVS was measured (Kennedy and Inglis, 2001, 2002; Ghanim et al., 2009; Lowrey and Bent, 2009; Matsugi et al., 2017) before and after crTMS and after nGVS (see Figure 1). The H-reflex indicates the excitability of the spinal motoneuron pool (Knikou, 2008). Short duration square-wave pulse GVS can alter the firing rate of primary vestibular neurons in a polarity-dependent manner (Goldberg et al., 1984), indicating that electrostimulation of the mastoid processes provides constant stimulation to vestibular neurons. Therefore, the modulation of the H-reflex by short duration square-wave pulse GVS reflects the excitability of the vestibulospinal response.

**FIGURE 4:**
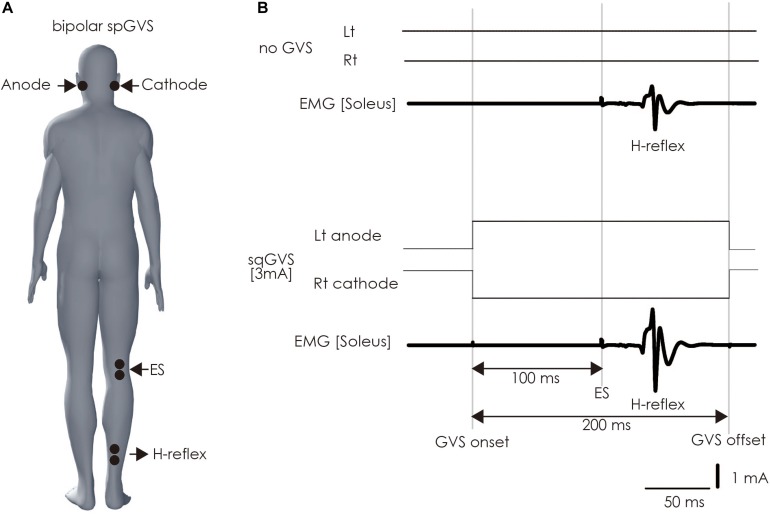
Method of assessing vestibulospinal function. **(A)** Electrode positioning. The electrode for the GVS was placed on bilateral mastoid processes. The electrodes were placed on the right soleus muscle to stimulate the tibial nerve to induce H-reflex therein and perform electromyography. **(B)** The sqGVS and typical H-reflex waveforms. The ES to induce the H-reflex was delivered 100 ms before sqGVS onset. GVS, galvanic vestibular stimulation; sqGVS, square-wave pulse galvanic vestibular stimulation; EMG, electromyography; ES, electrical stimulation.

The participant lay down on a bed in the prone position with his or her eyes closed, right and left ankle joints fixed at 90 degrees, and with braces to prevent unwanted movement of ankle joints. A bipolar binaural square-wave pulse GVS was delivered via Ag/AgCl surface electrodes affixed to the mastoid processes (Britton et al., 1993; Fitzpatrick et al., 1994; Welgampola and Colebatch, 2001; Ghanim et al., 2009; Lowrey and Bent, 2009; Matsugi et al., 2017) (right, cathode; left, anode; Figure 4). The GVS consisted of a 200-ms square-wave pulse that was delivered using an electrical isolator (SS-104J, Nihon Kohden, Japan) driven by a stimulator (SEN-3301, Nihon Kohden, Japan); the intensity was set at 3 mA (Fitzpatrick and Day, 2004; Okada et al., 2018).

Electromyography signals used to measure the H-reflex were recorded as previously described (Matsugi et al., 2017). Two Ag/AgCl surface-recording electrodes were placed 2 cm apart on the right soleus muscle. The EMG signals were amplified using an amplifier (MEG-1200, Nihon Kohden, Japan) with a pass-band filter of 15 Hz to 3 kHz. The EMG signals were converted to digital signals at a sampling rate of 10 kHz using an A/D converter (PowerLab 800S, AD Instruments; AD Instruments, Colorado Springs, CO, United States). The digital signals were then stored on a personal computer.

We delivered electrical stimulation (ES) to the right tibial nerve to evoke the H-reflex in the right soleus muscle 100 ms after the onset of the short duration square-wave pulse GVS (Kennedy and Inglis, 2001; Ghanim et al., 2009; Matsugi et al., 2017) (Figure 4). The H-reflex is reportedly facilitated by cathodal GVS in the inter-stimulus interval (Ghanim et al., 2009). The maximal M-wave (M-max) was measured at the beginning of all of the experimental trials, and the test for the right soleus H-reflex amplitude was periodically adjusted to a level 15–25% of the M-max during the experiment to adjust for the ascending limb of the H-reflex recruitment curve (Crone et al., 1990; Matsugi et al., 2017). Ten H-reflexes were elicited and recorded in the non-GVS condition (as control). In the right cathodal sqGVS condition, the two trials were performed in a random order, and the interval between tests was set to >7 s.

Before the test, we confirmed that the sqGVS did not produce sensations of pain or phosphine behind the eyes but did prompt body sway to the anodal side in participants standing with their eyes closed, feet together, and head facing forward (Fitzpatrick and Day, 2004). Furthermore, the GVS response test was conducted more than four times to ensure the participants were habituated to the sqGVS before test trials; although the first GVS responses were larger than the fifth, there was no subsequent change after the fifth trial (Balter et al., 2004).

### Analysis

H-reflex amplitude and M-wave amplitude in individual wave form was measured, and H-reflex as a percent of M-max amplitude was calculated in all trials and examinations based on the formula: H-reflex amplitude/M-max amplitude × 100. M-wave a percent of M-max amplitude was similarly calculated: M-wave amplitude/M-max amplitude × 100. To estimate the excitability of vestibulospinal function, the H-reflex ratio was calculated as the conditioned H-reflex amplitude/unconditioned H-reflex amplitude (Matsugi et al., 2017; Matsugi and Okada, 2020).

To test the baseline stimulation is equal, the paired sample test was conducted. If test of normality (Shapiro–Wilk test) revealed that normality of data, *t*-test was used. If in not normality of data, Wilcoxon test was used.

To test the effect of intervention of crTMS or nGVS (Sham and Real) and time (Pre- and Post-stimulation) on the H-reflex ratio, two-way analysis of variance (TW-ANOVA) was used if equality of variances was confirmed by Levene’s test. When a main effect was observed on these parameters, *post hoc* comparison (*t*-test) was conducted. When an interaction effect was observed on the means of H-reflex ratios, the *post hoc* comparison (*t*-test) was conducted to detect significant differences between groups.

In Analysis 1, to estimate the effect of crTMS on excitability of the vestibulospinal response, the H-reflex ratios obtained from pre-crTMS and post-crTMS in the real- and sham-crTMS conditions were analyzed (Figure 1). In Analysis 2, to gauge the effect of nGVS on the excitability of the vestibulospinal response, the H-reflex ratios obtained from the four pre- and post-nGVS trials performed after the sham-crTMS were analyzed (Figure 1). In analysis 3, to estimate the effect of cerebellar crTMS on the effect of nGVS, the H-reflex ratios obtained from the pre- and post-nGVS trials performed after the real-crTMS were analyzed (Figure 1).

The alpha level was set at 0.05 in all statistical analyses. Statistical analyses were conducted with using R software (version 3.1.2; the R Foundation for Statistical Computing, Vienna, Austria).

*Post hoc* power analysis was conducted to estimate the power (1 – beta error probability) for conducting Wilcoxon signed rank sum tests to compare the smallest groups (sham-nGVS in post-real-crTMS, *n* = 12) with software G^∗^power 3.1 (Version 3.1.9.4) provided by Faul et al. (2007).

## Results

None of the participants experienced any harmful side effects attributable to any of the examinations. As four participants were unable to endure the sqGVS before the examination, the examinations were terminated for these participants. Fifty-nine participants responded to the sqGVS while standing by engaging in a body sway to the anodal side (Fitzpatrick and Day, 2004), and no participant reported to sensation to nGVS.

**FIGURE 5:**
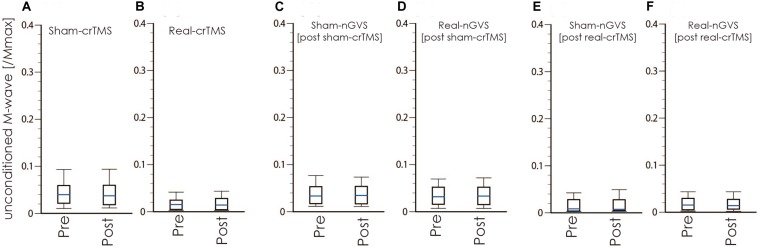
Boxplots of the M-wave amplitude in crTMS **(A,B)**, post-sham-crTMS **(C,D)**, and post-real-crTMS **(E,F)**. The middle horizontal lines indicate the median; the top and bottom lines of the box indicate the third and first quartiles, respectively; and the top and bottom vertical lines indicate the 90th and 10th percentiles, respectively. nGVS, noisy galvanic vestibular stimulation; crTMS, cerebellar repetitive transcranial magnetic stimulation.

**FIGURE 6:**
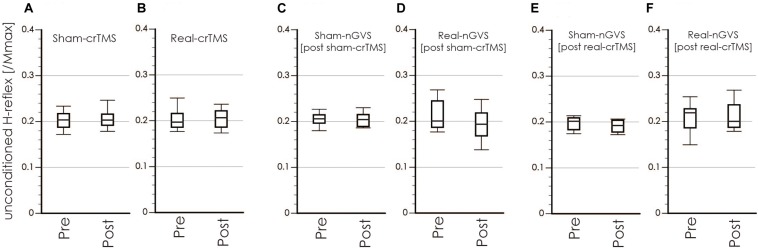
Boxplots of the unconditioned H-reflex amplitude in crTMS **(A,B)**, post-sham-crTMS **(C,D)**, and post-real-crTMS **(E,F)**. The middle horizontal lines indicate the median; the top and bottom lines of the box indicate the third and first quartiles, respectively; and the top and bottom vertical lines indicate the 90th and 10th percentiles, respectively. nGVS, noisy galvanic vestibular stimulation; crTMS, cerebellar repetitive transcranial magnetic stimulation.

Tables 1, 2 show the results of Shapiro–Wilk test, and paired sample test in M-wave and unconditioned H-reflex amplitude (Figures 5, 6). These results indicate the there was no significant difference between stimulation conditioned.

**TABLE 1:** Paired Samples *T*-Test (unconditioned M-wave).

		**Test of normality**								**95% CI for**	
		**(Shapiro-Wilk)**								**effect size**	
		**W**	**p**	**Test**	**Statistic**	**df**	**p**	VS-MPR*	**Effect Size**	**Lower**	**Upper**
Analysis 1	Sham	0.942	0.091	**Student**	**1.939**	**30**	**0.062**	**2.135**	**0.348**	**−0.017**	**0.708**
				Wilcoxon	322		0.152	1.286	0.298	−0.095	0.611
	Real	0.708	<0.001	Student	−1.259	27	0.219	1.107	−0.238	−0.612	0.14
				**Wilcoxon**	**177**		**0.782**	**1**	**−0.128**	**−0.503**	**0.287**
Analysis 2	Sham	0.931	0.228	**Student**	**−1.552**	**16**	**0.14**	**1.335**	**−0.376**	**−0.864**	**0.122**
				Wilcoxon	56		0.353	1.001	−0.268	−0.673	0.26
	Real	0.836	0.014	Student	0.458	13	0.655	1	0.122	−0.406	0.646
				**Wilcoxon**	**62**		**0.583**	**1**	**0.181**	**−0.39**	**0.651**
Analysis 3	Sham	0.7	<0.001	Student	−1.215	11	0.25	1.062	−0.351	−0.927	0.241
				**Wilcoxon**	**30**		**0.519**	**1**	**−0.231**	**−0.704**	**0.385**
	Real	0.818	0.005	Student	1.045	15	0.313	1.012	0.261	−0.242	0.756
				**Wilcoxon**	**84**		**0.433**	**1**	**0.235**	**−0.307**	**0.662**

*Vovk-Sellke Maximum p-ratio: based on the p -value, the maximum possible odds in favor of H_1_ over H_0_ equals 1/[−e *p* log(*p*)] for *p* ≤ 0.37 (Sellke et al., 2001). For the Student t-test, effect size is given by Cohen’s *d*; for the Wilcoxon test, effect size is given by the matched rank biserial correlation. Significant results suggest a deviation from normality inTest of Normality (Shapiro-Wilk). Bolded values/terms indicate accepted results.

**TABLE 2:** Paired samples *T*-Test (unconditioned H-reflex).

		**Test of normality**								**95% CI for**	
		**(Shapiro-Wilk)**								**effect size**	
		**W**	**p**	**Test**	**Statistic**	**df**	**p**	VS-MPR*	**Effect Size**	**Lower**	**Upper**
Analysis 1	Sham	0.975	0.652	**Student**	**−1.547**	**30**	**0.132**	**1.374**	**−0.278**	**−0.634**	**0.083**
				Wilcoxon	150		0.056	2.278	−0.395	−0.676	−0.015
	Real	0.958	0.318	**Student**	**−0.408**	**27**	**0.687**	**1**	**−0.077**	**−0.447**	**0.295**
				Wilcoxon	194		0.849	1	−0.044	−0.437	0.362
Analysis 2	Sham	0.969	0.8	**Student**	**−0.011**	**16**	**0.992**	**1**	**−0.003**	**−0.478**	**0.473**
				Wilcoxon	80		0.89	1	0.046	−0.459	0.528
	Real	0.563	<0.001	Student	1.226	13	0.242	1.071	0.328	−0.217	0.86
				**Wilcoxon**	**74**		**0.194**	**1.157**	**0.41**	**−0.158**	**0.774**
Analysis 3	Sham	0.92	0.285	**Student**	**1.34**	**11**	**0.207**	**1.128**	**0.387**	**−0.209**	**0.967**
				Wilcoxon	51		0.38	1	0.308	−0.312	0.744
	Real	0.941	0.36	**Student**	**0.054**	**15**	**0.958**	**1**	**0.013**	**−0.477**	**0.503**
				Wilcoxon	66		0.94	1	−0.029	−0.528	0.484

*Vovk-Sellke Maximum p-ratio: based on the p -value, the maximum possible odds in favor of H_1_ over H_0_ equals 1/[−e *p* log(*p*)] for *p* ≤ 0.37 (Sellke et al., 2001). For the Student t-test, effect size is given by Cohen’s *d*; for the Wilcoxon test, effect size is given by the matched rank biserial correlation. Significant results suggest a deviation from normality inTest of Normality (Shapiro-Wilk). Bolded values/terms indicate accepted results.

**FIGURE 7:**
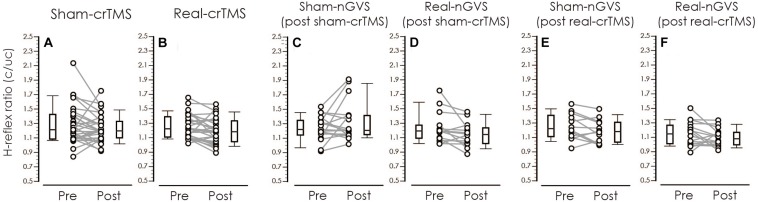
Boxplots of the H-reflex ratio (conditioned/unconditioned H-reflex amplitude) in crTMS **(A,B)**, post-sham-crTMS **(C,D)**, and post-real-crTMS **(E,F)**. The middle horizontal lines indicate the median; the top and bottom lines of the box indicate the third and first quartiles, respectively; and the top and bottom vertical lines indicate the 90th and 10th percentiles, respectively. The circles indicate the values of individual participants, and the gray lines connect the pre- and post-circles. There were significant reductions of the H-reflex ratio of post-real-crTMS **(B)** and of real-nGVS in post-sham-crTMS **(D)**. nGVS, noisy galvanic vestibular stimulation; crTMS, cerebellar repetitive transcranial magnetic stimulation.

Table 3 shows result of Levene’s test, and this test revealed that all parameters had equal variances for TW-ANOVA. These results indicate that there were equality of data and we can accept the result of parametric TW-ANOVA. Table 4 shows the For the H-reflex ratio in analyses 1 and 2, there was no significant effect of intervention and time, but in analysis 3, there was a significant main effect of intervention and no significant effect of time on the H-reflex ratio (Figure 7).

**TABLE 3:** Test for equality of variances (Levene’s) for two-way ANOVA.

	***F***	**df1**	**df2**	***p***	VS-MPR*
Analysis 1	2	3	114	0.118	1.459
Analysis 2	1.428	3	58	0.244	1.069
Analysis 3	0.967	3	52	0.415	1

*Vovk-Sellke maximum p-ratio: based on the p-value, the maximum possible odds in favor of H_1_ over H_0_ equals 1/[−e p log(p)] for p ≤ 0.37 (Sellke et al., 2001).

**TABLE 4:** ANOVA.

	**Cases**	**Sum of squares**	**df**	**Mean square**	**F**	**p**	VS-MPR*	**η^2^**
Analysis 1	CS	0.049	1	0.049	1.127	0.291	1.024	0.01
	Time	0.091	1	0.091	2.111	0.149	1.297	0.018
	CS * Time	4.388e-4	1	4.388e-4	0.01	0.92	1	0
	Residual	4.911	114	0.043				
Analysis 2	CS	0.14	1	0.14	3.093	0.084	1.769	0.048
	Time	5.538e-5	1	5.538e-5	0.001	0.972	1	0
	CS * Time	0.134	1	0.134	2.953	0.091	1.686	0.046
	Residual	2.634	58	0.045				
Analysis 3	CS	0.046	1	0.046	1.85	0.18	1.193	0.031
	Time	0.136	1	0.136	5.489	**0.023**	4.241	0.092
	CS * Time	2.745e-4	1	2.745e-4	0.011	0.917	1	0
	Residual	1.29	52	0.025				

Type III Sum of Squares. CS, conditioning stimulation, *Vovk-Sellke Maximum p-ratio: based on the p -value, the maximum possible odds in favor of H_1_ over H_0_ equals 1/[−e *p* log(*p*)] for *p* = 0.37 (Sellke et al., 2001). Bolded value indicates significant.

The *post hoc* power analysis revealed that the effect degree was 3, as calculated using the mean and standard deviation difference in this group were 0.06 and 0.02, respectively. Further, the input parameters were set as alpha error = 0.05 and sample size = 12, resulting in a power (1 – beta error probability) of 1.

## Discussion

The present study aimed to investigate whether crTMS and nGVS modulate the excitability of vestibulospinal function. An indicator of vestibulospinal response excitability, the H-reflex ratio was not significantly changed by real- or sham-crTMS (first analysis) or by real- or sham-nGVS (second analysis). On the other hand, our third analysis revealed a significant main effect of nGVS on the H-reflex ratio after real-crTMS. These findings indicate that crTMS alone and nGVS alone cannot affect excitability of the vestibulospinal response in participants in the static prone position; however, the nGVS effect could be observed after crTMS. These new findings suggest that crTMS facilitates the effect of nGVS on vestibulospinal function.

Our first analysis revealed that real- and sham-crTMS had no significant effect on the H-reflex ratio, indicating that crTMS does not directly affect excitability of the vestibulospinal response. A previous study showed that low-frequency crTMS reduces CBI, but does not change the excitability of contralateral M1 (Popa et al., 2010), indicating that the low-frequency crTMS disinhibits the excitability of the cerebellar output, but that the stimulation cannot directly affect excitability of remote brain sites. Therefore, in this study, the excitability of vestibular nuclei in the brainstem could not have been directly changed by crTMS alone.

Our secondary analysis showed that the H-reflex ratio was not changed by nGVS after sham-crTMS. This finding suggests the effect of nGVS alone, because sham-crTMS should not affect the cerebellar and vestibular function. Therefore, this result indicates that the application of nGVS alone cannot affect excitability of the vestibulospinal response of a healthy population in the static prone position. Moreover, nGVS modulates the threshold of the motor response through vestibular input and improves body balance in standing humans (Fujimoto et al., 2016, 2018; Wuehr et al., 2017; Inukai et al., 2018). The stochastic resonance and noise addition to non-linear systems inducing the change in plasticity of information processing in neural systems may account for these findings (McDonnell and Ward, 2011). On the other hand, the effect was obtained only in participants with large body sway during upright standing and no effect in participant with small body sway in young adult populations (Inukai et al., 2018). Therefore, in the present study, the effect of nGVS on vestibulospinal function in participants in a static prone position may be small compared to that of participants in an unstable position. As a result, we may have failed to discover an effect of nGVS alone on excitability of the vestibulospinal response in participants in a static prone position.

Our third analysis showed a significant main effect of nGVS on excitability of the vestibulospinal response after real-crTMS. The following mechanisms may account for the facilitation of the nGVS effect after crTMS. The modulation of vestibular reflex is affected by the cerebellum (Jang and Kim, 2019). The cerebellum contributes to adaptive changes in the vestibulo-ocular reflex, as shown by the observation that cerebellar lesions disturb long-term adaptive changes in the vestibular reflex (Miles and Eighmy, 1980; Ito, 1998). In a previous study, low-frequency crTMS applied as a pre-conditioning stimulation could not immediately change the vestibulo-ocular movement, but affected the trainability of vestibulo-ocular movement for dynamic gazing (Matsugi et al., 2019). Therefore, in the present study, using the same stimulation paradigm, the change of cerebellar activity induced by crTMS may affect the susceptibility of vestibulospinal response excitability in response to nGVS.

Balance function was not measured in this study, because body movement may affect H-reflex excitability (Knikou, 2008). Therefore, the effect of crTMS and/or nGVS on balance function in positions such as the standing position should be further investigated. As to the question of whether TMS, applied using a butterfly coil, can induce an electrical field on the cerebellar structure, our simulation using SimNIBS suggests that the electrical field, induced during TMS using the butterfly coil, was localized to the cerebellum. Furthermore, Popa reported that crTMS, performed using a figure-eight coil at an angle of 180° to the coil surface, could modulate the excitability of cerebellar output measured by CBI (Popa et al., 2010). Considered alongside our results, these findings suggest that crTMS applied with a butterfly coil could stimulate the cerebellum. Nevertheless, it is difficult to fully guarantee that deep cerebellar tissue has been stimulated. Recently it was reported that deep brain TMS using H-coil can stimulate the deep brain areas (Roth et al., 2014; Zibman et al., 2019). Therefore, we should conduct future study using H-coil for more certainly stimulating the deep cerebellar tissue in this study design to make sure that our result is due to cerebellar stimulation. Another consideration was the small sample size in the sham-nGVS in post-real-crTMS group. However, our *post hoc* power analysis revealed a power value of 1, which is larger than the 0.8 value of the reference study (Faul et al., 2007); accordingly, this analytic power is sufficient to conduct Wilcoxon signed rank sum tests, even in the smallest group.

## Conclusion

Low-frequency crTMS alone and nGVS alone were insufficient to modulate excitability of the vestibulospinal response in a young population in the static prone position. In contrast, an effect of nGVS on vestibular function was obtained after crTMS. These findings suggest that the cerebellum modulates vestibulospinal function. Further clinical studies are required to investigate the effect of crTMS and nGVS in patients with vestibulospinal dysfunction.

## Data Availability Statement

The datasets generated for this study are available on request to the corresponding author.

## Ethics Statement

The studies involving human participants were reviewed and approved by ethics committee of Shijonawate Gakuen University. The patients/participants provided their written informed consent to participate in this study.

## Author Contributions

AM, YO, and NY initially designed this study. Experimental equipment for magnetic stimulation was provided by KH and YS, while AM, NY, and SN provided other equipment. The experiments were conducted by AM, SD, RH, and NM (SD and RH were blinded assessors). Data analyses were conducted by AM, and NM. AM conducted a simulation of the distribution of E-fields induced by TMS. AM initially wrote the manuscript. All authors revised the manuscript.

## Conflict of Interest

The authors declare that the research was conducted in the absence of any commercial or financial relationships that could be construed as a potential conflict of interest.
